# Comparative efficacy and safety of complementary and alternative therapies for tubal obstructive infertility: A protocol for network meta-analysis

**DOI:** 10.1097/MD.0000000000024810

**Published:** 2021-02-19

**Authors:** Shuang-Qian Dong, Xing-Long Zhao, Ying Sun, Jian-Wei Zhang

**Affiliations:** a Shandong University of Traditional Chinese Medicine, Jinan; b Tai’an Hospital of Traditional Chinese Medicine, Tai’an; c The Affiliated Hospital of Shandong University of Traditional Chinese Medicine, Jinan, Shandong Province, China.

**Keywords:** complementary and alternative therapies, network meta-analysis, protocol, tubal obstructive infertility

## Abstract

**Background:**

Infertility is a kind of global disease. Fallopian tubal obstruction is one of the most important causes of female infertility. Complementary and alternative therapies are effective in treating tubal obstructive infertility, but there is no study on a comprehensive comparison among them. So, the purpose of this paper is to evaluate the efficacy and safety of different complementary and alternative therapies for tubal obstructive infertility.

**Methods:**

We will search for randomized controlled trials (RCTs) from the following databases: PubMed, Cochrane Library, EMBASE, Web of Science, Chinese Biomedical Literature Database (SinoMed), Chinese National Knowledge Infrastructure (CNKI), Wanfang database, and VIP database. We will assess the risk of bias of the included studies with the Cochrane tool, and the strength of evidence with the GRADE approach. Both pairwise meta-analyses and network meta-analyses will be performed to examine the relative efficacy and safety of complementary and alternative therapies in the treatment of tubal obstructive infertility.

**Conclusion:**

Our findings will provide clear evidence based on current available studies, which may lead to some proposals for both patients and researchers.

**INPLASY registration number:**

INPLASY202110076.

## 1 Introduction

Infertility is a reproductive system disease defined as failure to achieve a clinical pregnancy after regular unprotected sexual intercourse for 1 year or more.^[1,2]^ In the 1990 s, a study by the World Health Organization (WHO) showed that prevalence of infertility in developed countries was about 5% to 8%, while in some parts of developing countries it was about 30%, and the number of infertility patients ranges from 80 million to 110 million worldwide.^[3]^

Environmental pollution, sexually transmitted diseases, work pressure, food safety and the increasing number of induced abortions result in the increase of incidence of infertility, which has a great impact on human health and development.^[4]^ Infertility will become the third largest disease in the 21st century predicted by the WHO, only after cancer and cardiovascular and cerebrovascular diseases.^[5]^ Now, infertility has become a worldwide reproductive health problem.^[6]^ Although infertility does not threaten life, it is directly related to personal physical and mental health, family stability, and even affects the sustainable development of society. With more and more couples postponing their childbearing plan,^[7,8]^ and the opening of the policy of 1 couple having 2 children in China, the proportion of pregnancies has increased.^[8,9]^ Fallopian Tubal obstruction is one of the most important causes of female infertility. The incidence of tubal obstruction was approximately 19% in women with primary infertility and approximately 29% in women with secondary infertility.^[10]^ Tubal obstruction can be caused by a wide range of etiologies, including infection and subsequent inflammation, tubal spasm, endometriosis, congenital abnormalities, fibrosis.^[11]^

With the development of assisted reproductive technology (ART), in vitro fertilization and embryo transfer (IVF-ET) has made great progress in the treatment of tubal obstructive infertility. At the same time, it also brings many complications such as multiple pregnancy, premature birth, birth defects, ovarian hyperstimulation syndrome (OHSS), high cost and ethical challenges.^[12–18]^

Traditional Chinese medicine has a long history in treating tubal obstructive infertility and complementary and alternative therapies are effective and can improve pregnancy rate. Many studies and system reviews have confirmed the clinical effect of complementary and alternative therapies for infertility. Complementary and alternative therapies that are widely used to treat tubal blockage include acupressure, retention of enema with Chinese medicine, moxibustion, et al. For example, Yue et al found that in the treatment of tubal obstructive infertility, the clinical pregnancy rate of the group using traditional Chinese medicine enema was significantly increased through a randomized controlled trial (RCT).^[19]^ A meta-analysis of acupuncture in the treatment of fallopian tube obstruction found that acupuncture has obvious advantages in the clinical application of tubal obstructive infertility and acupuncture can significantly increase tubal patency and pregnancy rate, and achieve better clinical therapeutic effect.^[20]^

There are so many complementary and alternative therapies for tubal obstructive infertility, but there is no study on a comprehensive comparison among them. So, we conducted this network meta-analysis (NMA) protocol to evaluate the efficacy and safety of different complementary and alternative therapies in the treatment of tubal obstructive infertility, hoping to provide comprehensive evidence.

## 2 Methods

This systematic review is registered on the International Platform of Registered Systematic Review and Meta-analysis Protocols (INPLASY) and the registration number is INPLASY202110076. We carry out our research program according to the Preferred Reporting Items for Systematic Review and Meta-Analysis Protocols (PRISMA-P) guidelines.^[21]^

### 2.1 Database and search strategy

The following electronic databases, PubMed, Cochrane Library, EMBASE, Web of Science, Chinese Biomedical Literature Database (SinoMed), Chinese National Knowledge Infrastructure (CNKI), Wanfang database, and VIP database will be searched from their inceptions to January 2021. We will establish search strategy in line with the instructions of the Cochrane handbook.^[22]^ Both academic papers and conference papers will be searched without date restrictions. The detailed search strategy for PubMed database is shown in Table 1.

**Table 1:** Detailed search strategy for PubMed.

No.	Search item
#1	infertility, female [MeSH Terms]
#2	female infertility [Title/Abstract] OR postpartum sterility [Title/Abstract] OR subfertility [Title/Abstract] OR female sterility [Title/Abstract] OR sub-fertility, female [Title/Abstract]
#3	#1 OR #2
#4	fallopian tube diseases [MeSH Terms]
#5	diseases, fallopian tube [Title/Abstract] OR tubal obstruction [Title/Abstract] OR obstruction, tubal [Title/Abstract] OR fallopian tubal obstruction [Title/Abstract]
#6	#4 OR #5
#7	complementary therapies [MeSH Terms]
#8	complementary medicine [Title/Abstract] OR alternative therapies [Title/Abstract] OR medicine, alternative [Title/Abstract] OR Chinese patent medicine [Title/Abstract] OR Complementary and alternative therapies [Title/Abstract] OR Chinese herbal drugs [Title/Abstract] OR Herbal Therapy [Title/Abstract] OR Therapies, Complementary [Title/Abstract]
#9	Chinese herbal medicines [Title/Abstract] OR acupuncture [Title/Abstract] OR moxibustion [Title/Abstract] OR herb-partitioned moxibustion [Title/ Abstract] OR medicine enema [Title/Abstract] OR TCM enema [Title/Abstract] OR external application with Chinese medicine [Title/Abstract] OR external application of TCM [Title/Abstract] OR ion-introduction therapy of TCM [Title/Abstract] OR retention of enema with Chinese medicine [Title/Abstract] OR herbal fumigation [Title/Abstract] OR Auricular acupressure [Title/Abstract]
#10	#7 OR #8 OR #9
#11	randomized controlled trial [Publication Type]
#12	randomized [Title/Abstract] OR random allocation [Title/Abstract]
#13	#11OR #12
#14	#3 AND #6 AND #10 AND #13

### 2.2 Inclusion criteria

#### 2.2.1 Research design

We will include only RCTs published in Chinese or English. Review papers, expert opinions and case reports will be excluded.

#### 2.2.2 Types of patients

Married women of childbearing age diagnosed with tubal obstructive infertility will be included. Patients with severe cardiovascular disease, cancer, endometriosis, ovarian dysfunction or polycystic ovary syndrome will be excluded. Race and duration of illness will not be restricted.

#### 2.2.3 Interventions

The experimental group should receive complementary and alternative therapies in combination with or without other treatments; if both the 2 groups receive surgical treatment, such as hysteroscopy combined with laparoscopy surgery or fallopian tube interventional recanalization surgery, the styles of operation must be the same.

#### 2.2.4 Outcomes measures

The primary outcomes will include the following measures:

1. Clinical total effective rate: (total effective number)/total number100%.
2. Clinical pregnancy rate: Clinical pregnancy is diagnosed on the basis of absence of menstruation and ultrasound. Clinical pregnancy rate= (clinical pregnancy number)/total number ×100%.
3. Tubal recanalization rate: Tubal recanalization is diagnosed on the basis of hysterosalpingography. (Tubal recanalization number)/total number ×100%.

The secondary outcomes are as follows:

1. Adverse reactions.
2. Quality of life.
3. Depression, anxiety or stress symptoms.

### 2.3 Study collection and data extraction

We will employ Endnote x8 software to classify and manage the literatures retrieved from the above databases. The repeated literature will be deleted. First of all, we will exclude the obviously irrelevant literatures by reading the abstract and title; then, the full text will be read for further checked; finally, 2 researchers will independently extract data from the included studies using standardized data extraction excel designed for this study. We will record the following data information:

1. Trial characteristics: author, title, date of publication, journal, random method, inclusion criteria and registration number.
2. Participant characteristics: age, duration of disease, diagnostic criteria and sample size.
3. Intervention details: course of treatment, ways of specific interventions, frequency and detailed outcome.
4. Others: adverse reactions, depression or anxiety.

Disagreement will be settled through discussion or by a third author.

### 2.4 Assessment of risk of bias

According to Cochrane risk assessment tool, 2 researchers will independently evaluate risk of bias in 7 domains of each eligible trial. These 7 domains include random sequence generation, allocation concealment, blinding of the participants and outcomes assessment, incomplete outcome date, selective outcome reporting, and other bias. Each domain will be categorized as “low risk” “high risk” or “unclear”.

### 2.5 Statistical analysis

#### 2.5.1 Pairwise meta-analyses

In this process, continuous data will be described by mean difference (MD) or standardized mean difference (SMD). Odds Ratio (OR) will be used for dichotomous data. The 95% credible interval (CI) will be calculated. We will use *I*^2^ test to assess statistical heterogeneity.

#### 2.5.2 Network meta-analyses

We will conduct NMAs to examine the comparative efficacy and safety of complementary and alternative therapies. Random-effects model will be used to compare the direct and indirect evidence. Win BUGS and Stata software will be employed to perform network meta-analysis. The surface under the cumulative ranking curve (SUCRA) and the mean ranks will be reported to get the treatment hierarchy. The higher the SUCRA value, the more likely it is to be the best intervention. If there is enough evidence, we will conduct subgroup analysis and sensitivity analysis. The following factors will be used in subgroup analysis: surgical treatment or not, course of treatment. In additional, we will perform sensitivity analysis for the primary outcomes by excluding studies with high risk of bias.

### 2.6 Publication bias assessment

The potential publication bias and small study effect will be analyzed using comparison-adjusted funnel plots.

### 2.7 Evidence quality assessment

We will use the Grading of Recommendations Assessment, Development and Evaluation (GRADE) framework to evaluate the quality of evidence.^[23]^ The quality of evidence is divided into the following 4 levels: very low quality, low quality, moderate quality, or high quality.

## 3 Discussion

Tubal obstruction is one of the important causes of female infertility. IVF-ET is a solution of infertility caused by blocked fallopian tubes, but it also causes many problems. Many studies have shown that complementary and alternative therapies are effective in the treatment of tubal obstructive infertility. Our study aims to provide the best evidence summary, and give us a better comprehending of the relative efficacy about complementary and alternative therapies. At the same time, through this study, we will conclude that which is the best complementary and alternative therapy for tubal obstructive infertility. Notwithstanding some limitations in our study, we hope the results of our analysis will help doctors and patients make better choices.

## Author contributions

**Conceptualization:** Shuang-Qian Dong, Jian-Wei Zhang.

**Data curation:** Shuang-Qian Dong, Xing-Long Zhao, Ying Sun.

**Formal analysis:** Xing-Long Zhao, Ying Sun.

**Methodology:** Shuang-Qian Dong, Xing-Long Zhao.

**Search strategy**: Shuangqian Dong, Ying Sun.

**Software:** Shuang-Qian Dong, Xing-Long Zhao, Ying Sun.

**Statistical analysis & software:** Shuangqian Dong, Xinglong Zhao, Ying Sun.

**Writing – original draft:** Shuang-Qian Dong.

**Writing – review & editing:** Shuang-Qian Dong, Jian-Wei Zhang.
